# Expression and Function of Organic Anion Transporting Polypeptides in the Human Brain: Physiological and Pharmacological Implications

**DOI:** 10.3390/pharmaceutics13060834

**Published:** 2021-06-04

**Authors:** Anima M. Schäfer, Henriette E. Meyer zu Schwabedissen, Markus Grube

**Affiliations:** 1 Biopharmacy, Department Pharmaceutical Sciences, University of Basel, Klingelbergstrasse 50, 4056 Basel, Switzerland; anima.schaefer@unibas.ch (A.M.S.); h.meyerzuschwabedissen@unibas.ch (H.E.M.z.S.); 2 Center of Drug Absorption and Transport (C_DAT), Department of Pharmacology, University Medicine of Greifswald, 17489 Greifswald, Germany

**Keywords:** brain, blood–brain barrier, OATP, expression, neurosteroids, substrates

## Abstract

The central nervous system (CNS) is an important pharmacological target, but it is very effectively protected by the blood–brain barrier (BBB), thereby impairing the efficacy of many potential active compounds as they are unable to cross this barrier. Among others, membranous efflux transporters like P-Glycoprotein are involved in the integrity of this barrier. In addition to these, however, uptake transporters have also been found to selectively uptake certain compounds into the CNS. These transporters are localized in the BBB as well as in neurons or in the choroid plexus. Among them, from a pharmacological point of view, representatives of the organic anion transporting polypeptides (OATPs) are of particular interest, as they mediate the cellular entry of a variety of different pharmaceutical compounds. Thus, OATPs in the BBB potentially offer the possibility of CNS targeting approaches. For these purposes, a profound understanding of the expression and localization of these transporters is crucial. This review therefore summarizes the current state of knowledge of the expression and localization of OATPs in the CNS, gives an overview of their possible physiological role, and outlines their possible pharmacological relevance using selected examples.

## 1. Introduction

Every living organism is in constant contact with its environment. One mechanism associated with this “contact” is the exchange realized in the selective uptake and release of molecules of versatile chemical properties. This is an important achievement of life, which is already realized on the cellular level. In more highly developed organisms, including humans, entire organ systems are specialized in selectively absorbing substances, while at the same time preventing or at least impeding the entry of potentially harmful molecules. This “sorting function” is realized in organs such as the intestine, the liver or the kidney, and involves different mechanisms. On the one hand, the body can selectively absorb substances, an example of which would be the absorption of amino acids or glucose in the intestine. In both cases, these are very polar molecules that do not cross the cell membrane by passive diffusion. The biological solution for their transit through membranes is the presence of selective pores/uptake transporters that allow facilitated, carrier-mediated diffusion. On the other hand, we are also exposed to toxins, which can cross the cell membrane by passive diffusion. A possible protective mechanism here is to throw these compounds right back out of the cell. This function is realized by membrane efflux transporters, which are capable of transporting their substrates against a concentration gradient in an energy-dependent manner. In addition to these transport-based systems, toxic substances can be subject to enzymatic conversion. This can neutralize them and/or make them accessible to transport processes. The interplay of transporters and enzymes is highly effective in protecting the body from environmental toxins and is additionally involved in the elimination of endogenous metabolites. However, it always represents a compromise between the necessary intake of essential nutrients and protection from potential toxins. Accordingly, it does not appear surprising that protection may not be complete. For this reason, structures requiring special protection are located behind further biological barriers. Examples are the blood–placenta barrier, the blood–testis barrier, and the blood–brain barrier (BBB). Here, again, membrane proteins including the efflux and uptake transporters play an essential role. At the molecular level, these transporters are, on the one hand, members of the ABC transporter family, which primarily govern cellular efflux, and, on the other hand, members of the solute carrier (SLC) superfamily, which facilitate cellular uptake processes.

**Figure 1:**
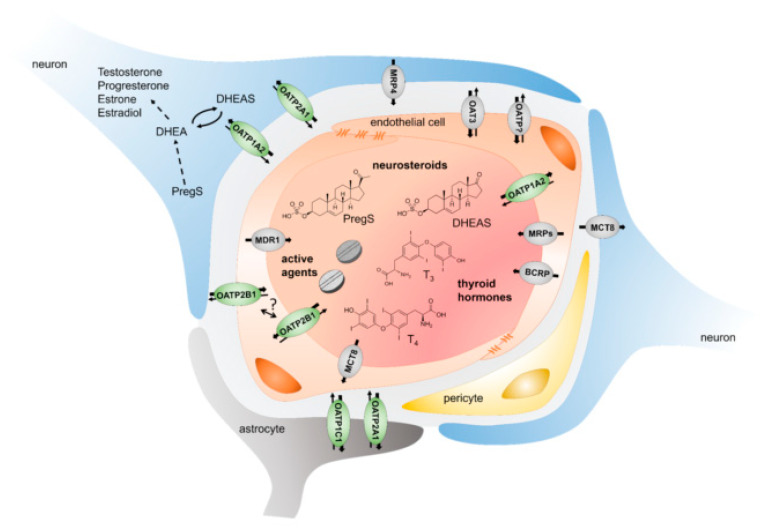
Schematic depicting the current understanding of uptake and efflux transporters at the blood–brain barrier. Uptake transporters enable the brain entry of molecules of endogenous (neurosteroids and thyroid hormones) or exogenous origin. Even if data on the localization of OATPs in the endothelial cells is sparse, OATP1A2 is assumed to be located at the apical membrane, while OATP2B1 might also be found at the basal membrane. Transcellular transport as well as protection involves efflux transporters such as MDR1 (ABCB1, P-Glycoprotein, P-gp), BCRP (ABCG2), and MRPs (ABCCs). Expression of OATP transporters and efflux transporters is also found in neurons and astrocytes enabling the movement of their substrates across these membranes. Adapted from with permission from [8], Biochem. Pharm. 2021.

From a pharmacological point of view, the expression of drug transporters in the central nervous system (CNS) is of particular interest, especially at the BBB. This cellular structure is considered practically impermeable for most drugs. While considered advantageous for most drug development programs, the protective capacity of the BBB becomes a major challenge in the development of neurotherapeutics [1]. In 2005, Pardridge concluded that 100% of the large molecule drugs and about 98% of the small molecule drugs currently approved are unable to cross the blood–brain barrier [2]. While this barrier function is absolute for many hydrophilic substances, lipophilic compounds are assumed to overcome the plasma membrane by means of passive diffusion. However, active transport mechanisms in the BBB lead to a more or less strong gradient between the systemic circulation and the brain, which in many cases does not allow pharmacologically sufficient concentrations in the CNS. This remarkable barrier function is linked to the extraordinary structure of the BBB. The endothelial cells contain highly dense tight junctions and adherens junction molecules, largely excluding the mechanisms of paracellular transport. In addition, fenestration, as found in the endothelial cells of the choroid plexus and other organs like the intestine, does not exist here and non-specific transcytosis (pinocytosis) is considered to be absent [3]. The integrity and impermeability of the BBB is further ensured by astrocytes and pericytes, as well as the basal lamina, which surrounds the endothelial cells [4]. For the astrocytes, it has been shown that they enhance the formation of tight junctions by secreting certain factors like sonic hedgehog [5]. In addition to these structural characteristics, the BBB also possesses active mechanisms that govern the exchange of molecules between the CNS and the blood, and therefore, the rest of the organism. These include membranous transporter proteins, both from the efflux and uptake transporter family. Representatives of the ABC transporter family, such as the P-glycoprotein (P-gp, MDR1, ABCB1), the breast cancer resistance protein (BCRP, ABCG2) or some multidrug resistance proteins (MRPs, ABCCs), are involved in the integrity of the BBB (compare Figure 1). These ATP-dependent transporters are mainly localized in the luminal membrane of the endothelial cells. The multiplicity of transporters and their high expression levels ensure that a wide range of molecules including those that exhibit the ability for passive diffusion are efficiently extruded back into the bloodstream, thereby limiting the accessibility of the CNS. In conclusion, ABC-transporters are major determinants of the fact that only a few drugs are able to cross the BBB. This is a phenomenon that has been worked on for years [6,7].

On the other hand, the CNS depends highly on the selective entry of certain compounds. Uptake transporters, and possibly also efflux transporters like MRP1 (ABCC1) localized in the abluminal membrane of the endothelial cells [9], are assumed to play a role in this context. The relevance of uptake mechanisms initiating centrally directed transport is exemplified by MCT8 (SLC16A2), where loss-of-function mutations in the *SLC16A2* gene lead to severe defects in neurological development due to reduced thyroid hormone CNS exposure [10,11]. Other relevant uptake transporters would be the L-type amino acid transporter 1 (LAT1, SLC7A5) with its substrate L-DOPA [12,13], or the glucose transporter GLUT1 (SLC2A1) [14], to name a few.

With the notion that facilitated BBB-endothelial entry could also realize central drug delivery, members of uptake and efflux transporter families recognizing pharmacological compounds as substrates are attracting interest in the field. Indeed, within the solute carrier (SLC) superfamily, pharmacologically important uptake transporters are found in the solute carrier organic anion (SLCO) family, among others. Members of this family are also known as organic anion transporting polypeptides (OATP). Accordingly, the *SLCO* nomenclature stands for the gene, while “OATP” denominates the respective protein. The aim of this review is to summarize the current state of knowledge regarding SLCO transporters in the CNS, especially at the blood–brain barrier, but also in the choroid plexus and neurons. Within this manuscript, we will address their expression and localization in these structures, but will also provide an overview of their endo- and exogenous substrates and discuss their known and potential physiological and pharmacological relevance.

## 2. OATP/SLCO Transporter

In humans, 11 SLCO transporters exist, organized into six families and ten subfamilies based on sequence identity. OATPs are predicted to have 12 transmembrane domains and act in a sodium-independent manner, thereby mediating the cellular uptake of a wide variety of endo- as well as exogenous compounds, including several clinically important drugs. In general, OATP substrates are amphipathic organic anions with a molecular weight of more than 300 Da; however, neutral or even positively charged substrates (especially for OATP1A2) have also been described. OATPs are involved in the uptake of several physiological substrates including organic anions like bile acids, bilirubin, thyroid hormones and prostaglandins [15]. Moreover, OATPs transport steroid hormone conjugates like estrone-3-sulfate or estradiol-17β-glucuronide. In general, more than 300 OATPs have been identified in several species, but in contrast to other transporter families, like the highly conserved ABC-transporter family, the SLCO-transporter family differs significantly among species. For example, while gene duplication resulted in the two human OATP1B- transporters, namely OATP1B1 and OATP1B3, the murine homolog for both transporters is OATP1B2 [15,16]. For OATP1A2, it is vice versa; here, we find only one transporter in the human system, while there are multiple OATP1A transporters in rodents [15]. These species differences are one reason for the difficulties in developing predictive animal models or in inferring potential effects in humans from animal data. Consequently, this is one of the reasons for which the physiological and pharmacological impact of OATPs in organs other than the liver is so far only poorly understood. Briefly, in the liver, a high clinical relevance was especially demonstrated for the SLCO-transporters OATP1B1 and OATP1B3. Both have been shown to be involved in the hepatic uptake of various drugs thereby influencing their systemic exposure. Importantly, the certainty in evaluating their clinical relevance is clearly driven by the fact that frequently occurring genetic loss-of-function variants exist, allowing in vivo comparisons for the impact of these transporters, which are mainly expressed in the liver [17,18,19,20]. In addition to the OATP1B- transporters, OATP1A2 and OATP2B1 have also been shown to interact with numerous drugs [21]; however, these findings are mainly from in vitro experiments and have so far only been validated to a very limited extent in vivo. In contrast to the OATP1B- transporters; however, OATP1A2 and OATP2B1 show a much broader expression profile, which is why they represent interesting candidates for drug uptake into their respective target structures [22,23].

Overall, the function of OATPs is of interest from both a physiological and pharmacological perspective. However, while it is relatively straightforward to determine the function of a transporter such as OATP1B1, which is expressed in only one organ and for which there are also functionally relevant genetic variants available, this is significantly more difficult for other OATPs. Particularly for those assumed to be of relevance for the CNS. Moreover, due to significant species differences, animal models are of limited value. Nevertheless, there are several ways to address this question. Each of these is based on a precise determination of the expression and localization of the transporter in a specific organ or tissue.

## 3. Expression and Localization of OATPs in the Brain

Within the following section, RNA expression data, antibody-based protein detection, which still plays an important role for localization, and modern mass spectrometry (MS)-based detection methods, are considered. Especially with regard to the latter method, it is important to emphasize that these methods not only target the whole brain, but also work with isolated microvessels or capillaries of the BBB and thus indirectly allow conclusions on localization.

### 3.1. OATP1A2

In 1998, in search of an uptake transporter for dehydroepiandrosterone-sulfate (DHEAS), the RNA of OATP1A2 was detected in the CNS of humans [24]. Subsequently, this finding was confirmed and validated on the protein level applying Western blot analysis of the whole brain homogenate [25]. Immunohistological staining indicates that the transporter is localized in brain capillaries and endothelial cells of the BBB [25,26,27]. Very recently, we were able to confirm this endothelial localization of OATP1A2; however, we also found an expression in glia cells indicated by the colocalization with the glial fibrillary acidic protein (GFAP) [28]. In a further study, OATP1A2 was also detected in neurons of the cortex, as well as pyramidal and granule cells of the hippocampus. Moreover, the authors also reported the presence of the transporter in the retina, which developmentally arises from an out pouch of the diencephalon and is thus part of the CNS [29]. Using targeted proteomic-based studies, OATP1A2 signals were found in whole brain lysates [28], while the transporter was below the detection limit in isolated brain capillaries [30,31]. Interestingly, using targeted proteomics, OATP1A2 was also below the detection limit in canine brain capillaries, while it was detected in the choroid plexus of dogs and rats, but not in that of humans [32,33]. Irrespective of the data obtained in the MS-based studies, we would consider OATP1A2 to be present in the cells forming the human blood–brain barrier especially in the endothelial cells.

### 3.2. OATP1C1

Already with their first description, the thyroid hormone (thyroxine (T_4_) and reverse triiodothyronine (rT_3_)) transporter, OATP1C1, was described in multiple regions of the human brain [34]. Further studies localized the transporter at the cellular level in astrocytes of the hippocampus and the hypothalamus [35,36,37]. Moreover, human OATP1C1 was detected in the apical and basal membrane of the ependymal cells of the choroid plexus, but only at low expression levels in microvessels of the BBB [36,38]. Similar observations were made in rodents concerning astrocytes and the choroid plexus; however, in these studies, OATP1C1 was also found in endothelial cells of the BBB [34,37,38]. The low expression of OATP1C1 in the human BBB was confirmed by further studies using targeted proteomics on microvessels isolated from the brain of humans and cynomolgus monkeys [30,39]. In both cases, OATP1C1 was below the detection limit, indicating no or only very low expression of OATP1C1 in the BBB of humans and monkeys. In summary, OATP1C1 is expressed in different regions of the human CNS mainly in astrocytes and the choroid plexus, but presumably not in the human BBB.

### 3.3. OATP2A1

OATP2A1, also known as prostaglandine transporter (PGT), is responsible for the transmembrane transport of prostaglandins. As assumed for other OATPs [8,40], OATP2A1 can act in both directions, as shown by its involvement in the release of prostaglandins [41]. Even if OATP2A1 function in the brain has only been of limited interest so far, its mRNA expression has already been shown in early studies for different brain areas such as the amygdala, the corpus callosum, the hippocampus, the substantia nigra, the thalamus [42], and ocular tissues [43]. On the protein level the transporter was found in neurons, microglia, and astrocytes [44] of the human brain, while in mice OATP2A1 was also present in the choroid plexus as well as in endothelial cells, microglia and perivascular cells of the cerebral cortex [45], but again targeted proteomic approaches so far have failed to verify these results in humans [30].

### 3.4. OATP2B1

Like OATP1A2, OATP2B1 shows a broad tissue distribution, which also includes the CNS [46]. Immunohistochemical stainings indicate that the transporter is localized in the endothelial cells of the human BBB [27,28,29] and the retina [29]. These results were confirmed in recent studies using targeted proteomic approaches for whole brain lysate [28] and enriched microvascular endothelial cells [47,48]. However, in some reports, OATP2B1 signals are below the detection limit even if a similar technique was applied [30,31]. Taken together, the current data indicate that in humans OATP2B1 is almost exclusively expressed in the BBB. This tissue distribution is in contrast to the situation in rodents, where the transporter was also present in the ependymal cells of the choroid plexus [9].

### 3.5. Other OATPs

Besides these transporters, other OATPs have been found in the human brain. One example is OATP3A1, which is involved in the prostaglandine and thyroid hormone uptake [49,50]. OATP3A1 exists in two splice variants, of which OATP3A1_v2 lacks the 18 C-terminal amino acids which are present in the OATP3A1_v1 [51]. Both variants are present in the brain. While OATP3A1_v1 was found in the grey, but not in the white matter, OATP3A1_v2 was present in neuronal cells of both structures. At the choroid plexus, OATP3A1_v1 was localized to the basal membrane and OATP3A1_v2 to the apical membrane of the ependymal cells [51]. The expression of OATP3A1 was also assessed using targeted proteomics, confirming its presence in the human choroid plexus [33], while its signal intensities were below the detection limit in human brain microvessels [30]. Of note, similar experiments using porcine brain capillaries demonstrated a predominately luminal (apical) expression of this transporter [52]. At this point, it should be mentioned that OATP3A1, unlike most other OATPs, shows high homology to other species such as rats, which makes animal models very interesting for studies investigating its function [50].

Finally, there are data on the thyroid hormone transporter OATP4A1 in the human brain [49]. Here, mRNA signals were detected in the cortex [53]; however, compared to other tissues its expression has to be considered low [54]. In rats, OATP4A1 was found to be present in the choroid plexus [55]; however, no data are currently available for humans. Like most other OATPs, OATP4A1 expression levels were assessed using targeted proteomics in human brain microvessels. Here, its signal intensities were below the detection limit [30], which is in line with an immunofluorescence study of OATP4A1 in the brain [27].

In summary, several OATPs have been detected in the human brain (compare Figure 1). While OATP1C1 and OATP2A1 are mainly of interest from a (patho)physiological point of view, OATP1A2 and OATP2B1, in addition to their possible importance in neurosteroid transport, could also be pharmacologically relevant due to their localization in the BBB and their substrate spectrum. However, especially with regard to the localization in the BBB, further investigations are necessary. The transporters were detected in this structure using different antibodies, but it is striking that these observations could often not be confirmed by means of targeted proteomics. These heterogeneous results could be explained by differences in the selection of peptides for the detection of the transporters, the sensitivity of the mass spectrometers, or the protocol used. It will be interesting to see whether this would still be true when using more sensitive instruments in the future. In addition, it is important to assess on which side of the endothelial cells the transporters are localized. Previous work assumes a luminal localization of both transporters [27], which would be rather unusual, at least with regard to OATP2B1, which is localized to the basolateral membrane in polarized cells of most other tissues like hepatocytes [46] or the placental syncythiotrophoblast [56]. Therefore, it would be of interest to have data from high-resolution microscopy. Another interesting approach to answer this question would be the targeted proteomics-based analysis of previously separated luminal and basal membranes of endothelial cells as already done for porcine brain capillaries [52].

## 4. Endogenous Substrates and the Physiological Role of OATPs in the Brain

It is generally accepted that OATPs are involved in the handling of sulfated steroid hormones. Estrone 3-sulfate (E_1_S), pregnenolone-sulfate (PregS), and dehydroepiandrosterone sulfate (DHEAS) are examples of conjugated steroids that depend on transport mechanisms to cross cellular membranes due to their hydrophilic sulfate moiety. Particularly for DHEAS and PregS, there are reports showing their effects on processes in the central nervous system [57,58,59]. Interestingly, the main action of these so-called neurosteroids is not seen in the regulation of gene transcription by interaction with steroid hormone receptors, but in the binding to and modulation of neuronal membrane receptors or ion channels [57,58]. In this context, neurosteroids have been shown to modulate neuronal excitability, for example, by interaction with GABA-A or NMDA receptors [60]. Notably, for the GABA-A receptor, it has been shown that PregS acts as a non-competitive antagonist, while unconjugated pregnenolone, inversely, agonizes the receptor [61]. Importantly, even if sulfated steroids are not themselves involved in the intracellular transcriptional regulation, they still function as precursors of the unconjugated and usually active steroids, which bind to their respective receptors. Both forms, the sulfated and desulfated steroids, are believed to exist in the central nervous system. Here, sulfation and desulfation, which are catalyzed by sulfotransferases and sulfatases, respectively, are the metabolic link between both forms of the steroids [62]. Moreover, it is believed that the steroids and/or their conjugated forms can be directly synthesized in the brain [63,64,65], or can be taken up from the blood stream. For pregnenolone, Kancheva et al. found significantly lower concentrations in the cerebrospinal fluid compared to serum, suggesting at least a diffusion-limiting barrier between the blood and the brain for this steroid. Importantly, in the same study, the authors observed a link between serum PregC (pregnenolone conjugates) and the unconjugated steroid in the CSF, while no such association was observed for pregnenolone in serum and CSF [66]. Referring to data by Wang et al. showing that intravenously applied pregnenolone sulfate can be found in various regions of the rat brain [67], Kancheva et al. concluded that the transfer of PregS from the blood to the brain should be considered as one of the mechanisms contributing to the homeostasis of this neurosteroid [66]. For DHEAS, there are data assuming both influx and efflux across the blood–brain barrier. Kancheva et al. found that the CSF DHEA depends more on the DHEA serum levels, suggesting that the situation for this steroid is somewhat different to that assumed for Pregnenolone [66]. Here, we want to cite Asabe et al., who observed a net efflux of DHEAS from the brain to the blood in rats. Using in vitro experiments, they were able to identify rat OATP2 (today Oatp1a4) and, due to ATP depletion, an efflux transporter to be involved in this process [68].

Irrespective of whether influx or efflux predominates, sulfated neurosteroids are hydrophilic molecules and therefore their handling within the blood–brain barrier depends on a properly functioning and coordinated transport system comprising cellular entry and efflux when transcellular passage has to be realized. Especially within the step of cellular entry, organic anion transporting polypeptides could play a role. As recently reviewed, there are two candidates that are expressed in the human endothelial cells of the BBB [69] and are capable of transporting sulfated steroids [70]. These candidates are OATP1A2 and OATP2B1. Since OATP1A2 was also shown to be expressed in neurons [29], the transporter might be involved in the neuronal uptake before intracellular desulfation of the sulfated steroid. Furthermore, addressing the above-mentioned study by Asabe et al., in the human system OATP2B1 and/or OATP1A2 would be possible candidates for cellular uptake from the brain. With BCRP (ABCG2), which is an efflux transporter localized at the apical side of endothelial cells and capable of extruding DHEAS, the flux of this neurosteroid out of the brain would be granted. Regarding the usage of neurosteroids as a therapeutic intervention, it seems noteworthy that they are discussed, for example, as modulators in mood disorders [61,64], thereby turning the transcellular transit and/or potential uptake mechanisms into a central question of such a treatment approach.

Another class of hormones that is dependent on transmembrane transport is the thyroid hormone family [71], which comprises thyroxine (T_4_) and the highly biologically active triiodothyronine (T_3_). The metabolism of thyroid hormones is rather complex; however, in the context of membrane transporters, it should be mentioned that one of the mechanisms contributing to the biological activity of thyroid hormones is the binding of T_3_ to respective intracellular receptors. T_4_ is linked to T_3_ by enzymes catalyzing the conversion by deiodination [72]. Independent of that, there are data supporting the idea that OATPs are involved in the transmembrane transport of thyroid hormones. One important OATP that transports thyroid hormones and is expressed in multiple regions of the human brain is OATP1C1 [34]. Even if there are reports suggesting low to no expression of OATP1C1 in the human blood–brain barrier [37,73], Sugiyama et al. were able to detect the rat orthologue OATP14 at the luminal and abluminal sides of rat brain capillary endothelial cells. Besides its expression, the authors reported on the thyroid hormone transport function of this particular orthologue in rats, showing high transport capacity for T_4_ and lower transport of T_3_ in OATP14-expressing HEK293 cells [74]. Notably, when performing uptake experiments using *X. laevis* oocytes injected with human OATP1C1, Pizzagalli et al. did not observe enhanced uptake of T_3_, but they were able to validate transport of T_4_ and showed transport of the biologically inactive reverse triiodothyronine (rT_3_) [34]. As mentioned before, thyroxine can be intracellularly converted to the more biologically active T_3_. This conversion is catalyzed by enzymes such as the type II deiodinase, which has been shown to be expressed in the human brain [75].

While OATP1C1 transport function seems to be linked to the precursor thyroxine, there are data obtained in a similar experimental set-up of injected *X. laevis* oocytes supporting that OATP1A2 mediates transport of both T_3_ and T_4_ (K_m_ T_3_: 6.5 ± 2.5 μM; T_4_: 8.0 ± 1.7 μM) [54]. For OATP2B1, the available data are controversial. While Kullack-Ublick et al. saw neither T_3_ or T_4_ accumulation in OATP2B1 expressing *X. laevis* oocytes [46], we provided data supporting the idea that both T_4_ and T_3_ interact with the transporter [76].

Besides function, one has to consider localization when speculating on the role of a transporter in the handling of a molecular class. In this context, we want to mention that it is currently not elucidated whether OATP2B1 is localized in the luminal (apical) or abluminal (basal) membrane of endothelial cells. However, at the abluminal site of the endothelial cells, OATP2B1 might play a role in the salvage of centrally formed sulfated thyroid hormone metabolites. Importantly, without going into too much detail, thyroid hormones undergo a complex metabolism of which some metabolites contain hydrophilic moieties [72]. For those metabolites, a transporter facilitating cellular entry would be of relevance. Further studies are needed to investigate the role of OATP2B1 and OATP1A2 in brain hormone homeostasis.

The importance of membrane transporters in the context of thyroid hormone homeostasis in the brain is exemplified by the Allan-Herndon-Dudley syndrome. This syndrome finds its genetic basis in mutations of the monocarboxylate transporter 8 (MCT8, *SLC16A2*), a well characterized thyroid hormone transporter, which is highly expressed in neurons and mainly transports T_3_ [75]. Patients deficient in MCT8 exhibit a disturbed thyroid hormone homeostasis in the brain, which leads to neonatal hypertonia with a total lack of posture acquisition, nystagmus, spasticity, dystonia, and profound mental retardation [77]. Besides its expression in neurons, MCT8 is also found in capillary endothelial cells, the choroid plexus and tanycytes, and is assumed to contribute to the CNS entry of thyroid hormones. Even if the role of membrane transport in thyroid hormone homeostasis is greatly supported by the findings in patients with Allan-Herndon-Dudley syndrome, little is known about the consequences of polymorphisms located within the gene loci encoding for other transporters assumed to be involved in thyroid hormone homeostasis. This is especially true for genetic variants of the herein described transporters OATP2B1 or OATP1A2. Notably, for OATP1C1, van der Deure et al. were not able to find an association between OATP1C1 polymorphisms (rs10770704, intron 3 T > C; rs36010656, c.427C > A, p.143P > T; rs10444412, c.3035C > T) and serum thyroid hormone parameters when investigating a large cohort of Danish twins [78]. However, there is a published report on a 15-year-old girl exhibiting neurological dysfunctions (development of dementia, spasticity, and intolerance to cold), where exome sequencing revealed a homozygous mutation in the *SLCO1C1* gene (NM_001145946.1:c.754G > A; p.(D252N)). Subsequent in vitro data revealed a loss in T_4_ transport function of this particular variant [79]. To further confirm this genetic association, it would be interesting to find more individuals harboring this mutation in order to validate the observation. Moreover, further data are needed to elucidate the consequences of the different affinities and substrate recognitions of the OATPs and the thyroid hormones and their metabolites, as it appears that a network of multiple transporters is involved in the homeostasis of thyroid hormones in the human brain.

## 5. Exogenous Substrates and the Pharmacological Role of OATPs in the Brain

Besides enabling the cellular entry of endogenous molecules, the OATPs were also shown to transport exogenous compounds thereby becoming pharmacokinetic determinants. OATP1B1, OATP1B3, OATP2B1 and OATP1A2 are best characterized for their interaction with drugs. In the context of pharmacology, OATP1B transporters need to be highlighted, as they are known to be critically involved in the hepatocellular uptake of their substrates. However, since they are only expressed in the liver, their relevance to the CNS is limited to their influence on the plasma concentration of their substrates. For the ubiquitously expressed OATP2B1, contributions to the ADME processes are believed to apply to various organs including intestine [80,81], liver [46], and kidney [82]. However, the relevance of OATP2B1 as a pharmacokinetic determinant is less defined than for the OATP1B-transporters. As mentioned above, OATP2B1 is not only present in the ADME organs, but also highly expressed in cells forming cellular barriers, such as the syncytiotrophoblasts of the blood–placenta barrier [83] or the endothelial cells of the BBB [28]. To date, there is not much data available regarding centrally active drugs and their interaction with OATP2B1. One reason might be the lack of specific inhibitors for each OATP, while another one might be the expression of OATP2B1 in other tissues, including the liver, making it difficult to differentiate between systemic effects and local ones (e.g., at the blood–brain barrier). Furthermore, due to its localization in the liver and BBB, an inhibition of OATP2B1 would cause opposing effects on plasma and tissue concentrations. In addition, to date, there are no frequent genetic loss-of-function variants, which could give information about the transporter’s contribution to physiological and pharmacological processes in comparative pharmacogenetic studies. Nevertheless, there was one approach investigating whether morphine and its two major metabolites [morphine-3- and morphine-6-glucuronide] rely on OATP2B1-transport to cross the blood–brain barrier [84]. In vitro experiments revealed that morphine and morphine-6-glucuronide are substrates of the transporter, while no difference in cellular accumulation was found for morphine-3-glucuronide when comparing OATP2B1-expressing and control cells. Interestingly, when blocking OATP2B1 in mouse brains using nanoparticles of DGL-PEG/dermorphin encaspulated siRNA (OATP2B1), Yang et al. observed less accumulation of morphine and morphine-6-glucuronide, translating into lower morphine tolerance and analgesic effect [84].

For OATP1A2, there have been in vitro experiments suggesting that this transporter may be—due to its expression in the BBB—involved in the brain entry of opioids. Briefly, using the *X. laevis* oocyte system, the naturally occurring linear opioid peptides deltorphin II and the synthetic cyclic peptide [D-penicillamine2,5] enkephalin (DPDPE) were identified as OATP1A2 substrates [25]. Another class of compounds known to enter the brain in order to exert its effect is dopamine receptor agonists. In our laboratory, we were able to show that both OATP2B1 and OATP1A2 interact with dopamine receptor agonists. Applying the method of competitive counterflow, we identified bromocriptine and cabergoline as substrates of the transporters [28]. It is beyond question that there are further studies needed to investigate the interaction of the transporters with the above-mentioned drugs, especially when it comes to the clinical relevance of the currently available data. Another class of molecules that could be interesting in this context is antitumor drugs, where multiple members have been reported to be transported by OATP1A2 and OATP2B1 [85].

One class of drugs that is widely accepted to be transported by multiple OATPs is the statins. These 3-Hydroxy-3-Methylglutaryl-Coenzyme-A-reductase inhibitors are used to treat dyslipidemia and to prevent cardiovascular events. The HMG-CoA reductase is the rate-limiting step in the mevalonate forming pathway. The end-products of this pathway are also involved in regulating biological activities in the central nervous system. As summarized by Fracassi and Marangoni et al., statins exhibit pleiotropic effects and have been shown to modulate a plethora of neuronal processes including the regulation of cognition and memory pathways [86]. Indeed, Johnson-Anuna et al. were able to show that the statins lovastatin, pravastatin, and simvastatin reach the mouse cerebral cortex by determining brain-statin levels using liquid chromatography/tandem mass spectrometry [87]. Both OATP2B1 and OATP1A2 recognize statins as substrates [88,89,90,91]. In the context of OATPs in the blood–brain barrier and their capacity to transport statins, we want to highlight the results of a preclinical study obtained in a mouse model of stroke, where the authors observed improved cerebral blood flow and the stabilization of neurological functions when administering simvastatin daily for 14 days before occlusion of the arteria cerebri media. Indeed, the authors observed reduced cerebral infarct size after simvastatin treatment when analyzing the tissue injury applying a staining method [92]. Whether there is an OATP-mediated uptake mechanism involved in the brain entry of the statin remains to be investigated. Notably, there is evidence that statins not only exert ameliorating effects on the CNS, including protection against neurodegenerative diseases, but also have a negative impact on cognitive functions in some patients. In a review on that specific topic, the authors conclude that evidence is insufficient; however, the available data suggest that some patients are more sensitive to experiencing reversible cognitive impairment including memory loss or confusion, while others profit from a reduced risk of dementia [93]. Whether changes in OATP2B1 and/or OATP1A2 transport activity impacts the observed effects can only be speculated on. However, Abdullahi et al. were able to show that the OATP1A2 rat orthologue OATP1A4 is involved in the CNS delivery of both hydrophilic (pravastatin) and hydrophobic (atorvastatin) statins using in situ brain perfusion technique [94]. Furthermore, using the same method, Ose et al. were able to show significantly decreased blood-to-brain transport of pitavastatin and rosuvastatin in OATP1A4 knock-out mice compared to wild-type mice [95]. Even if one certainly has to consider species-specific differences, these data support the idea of OATPs playing a role in the blood brain-barrier transfer of statins in humans. However, one has to keep in mind that we are speculating on this without relating the information on CNS statin exposure to the exposure in plasma or other tissues. Citing one of the excellent reviewers of this manuscript, it would be more relevant to compare the free drug exposure in plasma to that in extensively perfused tissues in order to comment on the role of drug transporters in the permeation of the BBB.

## 6. Conclusions

In summary, several OATPs have been detected in the human brain so far. Based on our current knowledge, OATP2B1 and OATP1A2 appear to be the most relevant drug transporters, besides the physiologically important thyroid hormone transporters. Both OATP2B1 and OATP1A2 are found in the blood–brain barrier; however, information on their exact localization is still limited. Considering the situation in other tissues, one would rather expect a basal localization for OATP2B1, whereas an apical one would be conceivable for OATP1A2. Thus, in both cases, but especially assuming a luminal localization, these transporters would be of potential interest for concepts of CNS-targeted drug delivery. However, so far there are only very limited data available demonstrating a clinically relevant role of these transporters for drug distribution into the CNS. Although it is known from in vitro studies that respective drugs are transported by these transporters, clinical studies on this topic are missing. This is not necessarily surprising, as the corresponding effects are very difficult to detect. For example, in contrast to OATP1B1, there are no frequently occurring genetic variants for either transporter that are associated with a high functional impairment. Even if interaction studies would be an alternative, there are currently no transporter-specific inhibitors available. Another possibility for addressing this problem would be an appropriate animal model. However, it must be kept in mind that OATPs and especially OATP1A2 show major species-specific differences. Therefore, humanized animal models may be an interesting alternative. Indeed, an appropriate humanized OATP1A2 mouse model has recently been published, but the data presented to date concerning an effect on drug uptake into the CNS are rather disappointing [96]. Another starting point could be the use of cellular models for the blood–brain barrier. Although the corresponding cell lines hardly express the mentioned membrane transporters, an overexpression would be conceivable here as well.

In conclusion, the pharmacological relevance and the pharmacological possibilities of OATPs in the blood–brain barrier are of great interest, but in many respects, we are still at the beginning of understanding their relevance.

## Data Availability

Not applicable.

## References

[1] Henderson J.T., Piquette-Miller M. (2015). Blood-brain barrier: An impediment to neuropharmaceuticals. Clin. Pharm. Ther..

[2] Pardridge W.M. (2005). The blood-brain barrier: Bottleneck in brain drug development. NeuroRx.

[3] Gawdi R., Emmady P.D. (2021). Physiology, Blood Brain Barrier. StatPearls.

[4] Dong X. (2018). Current Strategies for Brain Drug Delivery. Theranostics.

[5] Tietz S., Engelhardt B. (2015). Brain barriers: Crosstalk between complex tight junctions and adherens junctions. J. Cell Biol..

[6] Loscher W., Gericke B. (2020). Novel Intrinsic Mechanisms of Active Drug Extrusion at the Blood-Brain Barrier: Potential Targets for Enhancing Drug Delivery to the Brain?. Pharmaceutics.

[7] Koehn L.M. (2020). ABC efflux transporters at blood-central nervous system barriers and their implications for treating spinal cord disorders. Neural Regen. Res..

[8] Kinzi J., Grube M., Meyer Zu Schwabedissen H.E. (2021). OATP2B1—The underrated member of the organic anion transporting polypeptide family of drug transporters?. Biochem. Pharm..

[9] Roberts L.M., Black D.S., Raman C., Woodford K., Zhou M., Haggerty J.E., Yan A.T., Cwirla S.E., Grindstaff K.K. (2008). Subcellular localization of transporters along the rat blood-brain barrier and blood-cerebral-spinal fluid barrier by in vivo biotinylation. Neuroscience.

[10] Bernal J., Feingold K.R., Anawalt B., Boyce A., Chrousos G., de Herder W.W., Dhatariya K., Dungan K., Grossman A., Hershman J.M., Hofland J. (2000). Thyroid Hormones in Brain Development and Function. Endotext.

[11] Friesema E.C., Grueters A., Biebermann H., Krude H., von Moers A., Reeser M., Barrett T.G., Mancilla E.E., Svensson J., Kester M.H. (2004). Association between mutations in a thyroid hormone transporter and severe X-linked psychomotor retardation. Lancet.

[12] Terasaki T., Hosoya K. (2001). Conditionally immortalized cell lines as a new in vitro model for the study of barrier functions. Biol. Pharm. Bull..

[13] Puris E., Gynther M., Auriola S., Huttunen K.M. (2020). L-Type amino acid transporter 1 as a target for drug delivery. Pharm. Res..

[14] Koepsell H. (2020). Glucose transporters in brain in health and disease. Pflug. Arch..

[15] Hagenbuch B., Stieger B. (2013). The SLCO (former SLC21) superfamily of transporters. Mol. Asp. Med..

[16] Meyer Zu Schwabedissen H.E., Ware J.A., Tirona R.G., Kim R.B. (2009). Identification, expression, and functional characterization of full-length and splice variants of murine organic anion transporting polypeptide 1b2. Mol. Pharm..

[17] Maeda K. (2015). Organic anion transporting polypeptide (OATP)1B1 and OATP1B3 as important regulators of the pharmacokinetics of substrate drugs. Biol. Pharm. Bull..

[18] Nozaki Y., Izumi S. (2020). Recent advances in preclinical in vitro approaches towards quantitative prediction of hepatic clearance and drug-drug interactions involving organic anion transporting polypeptide (OATP) 1B transporters. Drug Metab. Pharm..

[19] Schwarz U.I., Meyer zu Schwabedissen H.E., Tirona R.G., Suzuki A., Leake B.F., Mokrab Y., Mizuguchi K., Ho R.H., Kim R.B. (2011). Identification of novel functional organic anion-transporting polypeptide 1B3 polymorphisms and assessment of substrate specificity. Pharm. Genom..

[20] Meyer Zu Schwabedissen H.E., Grube M., Kroemer H.K., Maitland-van der Zee A.-H., Daly A.K. (2012). Pharmacogenetics of Drug Transporters. Pharmacogenetics and Individualized Therapy.

[21] Roth M., Obaidat A., Hagenbuch B. (2012). OATPs, OATs and OCTs: The organic anion and cation transporters of the SLCO and SLC22A gene superfamilies. Br. J. Pharm..

[22] Zhou Y., Yuan J., Li Z., Wang Z., Cheng D., Du Y., Li W., Kan Q., Zhang W. (2015). Genetic polymorphisms and function of the organic anion-transporting polypeptide 1A2 and its clinical relevance in drug disposition. Pharmacology.

[23] McFeely S.J., Wu L., Ritchie T.K., Unadkat J. (2019). Organic anion transporting polypeptide 2B1—More than a glass-full of drug interactions. Pharm. Ther..

[24] Kullak-Ublick G.A., Fisch T., Oswald M., Hagenbuch B., Meier P.J., Beuers U., Paumgartner G. (1998). Dehydroepiandrosterone sulfate (DHEAS): Identification of a carrier protein in human liver and brain. FEBS Lett..

[25] Gao B., Hagenbuch B., Kullak-Ublick G.A., Benke D., Aguzzi A., Meier P.J. (2000). Organic anion-transporting polypeptides mediate transport of opioid peptides across blood-brain barrier. J. Pharm. Exp. Ther..

[26] Lee W., Glaeser H., Smith L.H., Roberts R.L., Moeckel G.W., Gervasini G., Leake B.F., Kim R.B. (2005). Polymorphisms in human organic anion-transporting polypeptide 1A2 (OATP1A2): Implications for altered drug disposition and central nervous system drug entry. J. Biol. Chem..

[27] Bronger H., Konig J., Kopplow K., Steiner H.H., Ahmadi R., Herold-Mende C., Keppler D., Nies A.T. (2005). ABCC drug efflux pumps and organic anion uptake transporters in human gliomas and the blood-tumor barrier. Cancer Res..

[28] Schäfer A.M., Meyer Zu Schwabedissen H.E., Bien-Moller S., Hubeny A., Vogelgesang S., Oswald S., Grube M. (2020). OATP1A2 and OATP2B1 Are Interacting with Dopamine-Receptor Agonists and Antagonists. Mol. Pharm..

[29] Gao B., Vavricka S.R., Meier P.J., Stieger B. (2015). Differential cellular expression of organic anion transporting peptides OATP1A2 and OATP2B1 in the human retina and brain: Implications for carrier-mediated transport of neuropeptides and neurosteriods in the CNS. Pflug. Arch..

[30] Uchida Y., Ohtsuki S., Katsukura Y., Ikeda C., Suzuki T., Kamiie J., Terasaki T. (2011). Quantitative targeted absolute proteomics of human blood-brain barrier transporters and receptors. J. Neurochem..

[31] Bao X., Wu J., Xie Y., Kim S., Michelhaugh S., Jiang J., Mittal S., Sanai N., Li J. (2020). Protein Expression and Functional Relevance of Efflux and Uptake Drug Transporters at the Blood-Brain Barrier of Human Brain and Glioblastoma. Clin. Pharm. Ther..

[32] Braun C., Sakamoto A., Fuchs H., Ishiguro N., Suzuki S., Cui Y., Klinder K., Watanabe M., Terasaki T., Sauer A. (2017). Quantification of Transporter and Receptor Proteins in Dog Brain Capillaries and Choroid Plexus: Relevance for the Distribution in Brain and CSF of Selected BCRP and P-gp Substrates. Mol. Pharm..

[33] Uchida Y., Zhang Z., Tachikawa M., Terasaki T. (2015). Quantitative targeted absolute proteomics of rat blood-cerebrospinal fluid barrier transporters: Comparison with a human specimen. J. Neurochem..

[34] Pizzagalli F., Hagenbuch B., Stieger B., Klenk U., Folkers G., Meier P.J. (2002). Identification of a novel human organic anion transporting polypeptide as a high affinity thyroxine transporter. Mol. Endocrinol..

[35] Schnell C., Shahmoradi A., Wichert S.P., Mayerl S., Hagos Y., Heuer H., Rossner M.J., Hulsmann S. (2015). The multispecific thyroid hormone transporter OATP1C1 mediates cell-specific sulforhodamine 101-labeling of hippocampal astrocytes. Brain Struct. Funct..

[36] Friesema E.C., Visser T.J., Borgers A.J., Kalsbeek A., Swaab D.F., Fliers E., Alkemade A. (2012). Thyroid hormone transporters and deiodinases in the developing human hypothalamus. Eur. J. Endocrinol..

[37] Alkemade A., Friesema E.C., Kalsbeek A., Swaab D.F., Visser T.J., Fliers E. (2011). Expression of thyroid hormone transporters in the human hypothalamus. J. Clin. Endocrinol. Metab..

[38] Roberts L.M., Woodford K., Zhou M., Black D.S., Haggerty J.E., Tate E.H., Grindstaff K.K., Mengesha W., Raman C., Zerangue N. (2008). Expression of the thyroid hormone transporters monocarboxylate transporter-8 (SLC16A2) and organic ion transporter-14 (SLCO1C1) at the blood-brain barrier. Endocrinology.

[39] Ito K., Uchida Y., Ohtsuki S., Aizawa S., Kawakami H., Katsukura Y., Kamiie J., Terasaki T. (2011). Quantitative membrane protein expression at the blood-brain barrier of adult and younger cynomolgus monkeys. J. Pharm. Sci..

[40] Schäfer A.M., Bock T., Meyer Zu Schwabedissen H.E. (2018). Establishment and Validation of Competitive Counterflow as a Method To Detect Substrates of the Organic Anion Transporting Polypeptide 2B1. Mol. Pharm..

[41] Kanai N., Lu R., Satriano J.A., Bao Y., Wolkoff A.W., Schuster V.L. (1995). Identification and characterization of a prostaglandin transporter. Science.

[42] Lu R., Kanai N., Bao Y., Schuster V.L. (1996). Cloning, in vitro expression, and tissue distribution of a human prostaglandin transporter cDNA(hPGT). J. Clin. Investig..

[43] Schuster V.L., Lu R., Coca-Prados M. (1997). The prostaglandin transporter is widely expressed in ocular tissues. Surv. Ophthalmol..

[44] Choi K., Zhuang H., Crain B., Dore S. (2008). Expression and localization of prostaglandin transporter in Alzheimer disease brains and age-matched controls. J Neuroimmunol..

[45] Nakamura Y., Nakanishi T., Shimada H., Shimizu J., Aotani R., Maruyama S., Higuchi K., Okura T., Deguchi Y., Tamai I. (2018). Prostaglandin Transporter OATP2A1/SLCO2A1 Is Essential for Body Temperature Regulation during Fever. J. Neurosci..

[46] Kullak-Ublick G.A., Ismair M.G., Stieger B., Landmann L., Huber R., Pizzagalli F., Fattinger K., Meier P.J., Hagenbuch B. (2001). Organic anion-transporting polypeptide B (OATP-B) and its functional comparison with three other OATPs of human liver. Gastroenterology.

[47] Billington S., Salphati L., Hop C., Chu X., Evers R., Burdette D., Rowbottom C., Lai Y., Xiao G., Humphreys W.G. (2019). Interindividual and Regional Variability in Drug Transporter Abundance at the Human Blood-Brain Barrier Measured by Quantitative Targeted Proteomics. Clin. Pharm. Ther..

[48] Ji C., Tschantz W.R., Pfeifer N.D., Ullah M., Sadagopan N. (2012). Development of a multiplex UPLC-MRM MS method for quantification of human membrane transport proteins OATP1B1, OATP1B3 and OATP2B1 in in vitro systems and tissues. Anal. Chim. Acta.

[49] Tamai I., Nezu J., Uchino H., Sai Y., Oku A., Shimane M., Tsuji A. (2000). Molecular identification and characterization of novel members of the human organic anion transporter (OATP) family. Biochem. Biophys. Res. Commun..

[50] Adachi H., Suzuki T., Abe M., Asano N., Mizutamari H., Tanemoto M., Nishio T., Onogawa T., Toyohara T., Kasai S. (2003). Molecular characterization of human and rat organic anion transporter OATP-D. Am. J. Physiol. Renal. Physiol..

[51] Huber R.D., Gao B., Sidler Pfandler M.A., Zhang-Fu W., Leuthold S., Hagenbuch B., Folkers G., Meier P.J., Stieger B. (2007). Characterization of two splice variants of human organic anion transporting polypeptide 3A1 isolated from human brain. Am. J. Physiol. Cell Physiol..

[52] Kubo Y., Ohtsuki S., Uchida Y., Terasaki T. (2015). Quantitative Determination of Luminal and Abluminal Membrane Distributions of Transporters in Porcine Brain Capillaries by Plasma Membrane Fractionation and Quantitative Targeted Proteomics. J. Pharm. Sci..

[53] Chan S.Y., Martin-Santos A., Loubiere L.S., Gonzalez A.M., Stieger B., Logan A., McCabe C.J., Franklyn J.A., Kilby M.D. (2011). The expression of thyroid hormone transporters in the human fetal cerebral cortex during early development and in N-Tera-2 neurodifferentiation. J. Physiol..

[54] Fujiwara K., Adachi H., Nishio T., Unno M., Tokui T., Okabe M., Onogawa T., Suzuki T., Asano N., Tanemoto M. (2001). Identification of thyroid hormone transporters in humans: Different molecules are involved in a tissue-specific manner. Endocrinology.

[55] Choudhuri S., Cherrington N.J., Li N., Klaassen C.D. (2003). Constitutive expression of various xenobiotic and endobiotic transporter mRNAs in the choroid plexus of rats. Drug Metab. Dispos..

[56] Grube M., Reuther S., Meyer Zu Schwabedissen H., Kock K., Draber K., Ritter C.A., Fusch C., Jedlitschky G., Kroemer H.K. (2007). Organic anion transporting polypeptide 2B1 and breast cancer resistance protein interact in the transepithelial transport of steroid sulfates in human placenta. Drug Metab. Dispos..

[57] Reddy D.S. (2010). Neurosteroids: Endogenous role in the human brain and therapeutic potentials. Prog. Brain. Res..

[58] Harteneck C. (2013). Pregnenolone sulfate: From steroid metabolite to TRP channel ligand. Molecules.

[59] Baulieu E.E. (1997). Neurosteroids: Of the nervous system, by the nervous system, for the nervous system. Recent Prog. Horm. Res..

[60] Ratner M.H., Kumaresan V., Farb D.H. (2019). Neurosteroid Actions in Memory and Neurologic/Neuropsychiatric Disorders. Front. Endocrinol..

[61] Reddy D.S., Kulkarni S.K. (2000). Development of neurosteroid-based novel psychotropic drugs. Prog. Med. Chem..

[62] Mueller J.W., Gilligan L.C., Idkowiak J., Arlt W., Foster P.A. (2015). The Regulation of Steroid Action by Sulfation and Desulfation. Endocr. Rev..

[63] Baulieu E.E. (1998). Neurosteroids: A novel function of the brain. Psychoneuroendocrinology.

[64] Maninger N., Wolkowitz O.M., Reus V.I., Epel E.S., Mellon S.H. (2009). Neurobiological and neuropsychiatric effects of dehydroepiandrosterone (DHEA) and DHEA sulfate (DHEAS). Front. Neuroendocr..

[65] Salman E.D., Faye-Petersen O., Falany C.N. (2011). Hydroxysteroid sulfotransferase 2B1b expression and localization in normal human brain. Horm. Mol. Biol. Clin. Investig..

[66] Kancheva R., Hill M., Novak Z., Chrastina J., Velikova M., Kancheva L., Riha I., Starka L. (2010). Peripheral neuroactive steroids may be as good as the steroids in the cerebrospinal fluid for the diagnostics of CNS disturbances. J. Steroid. Biochem. Mol. Biol..

[67] Wang M.D., Wahlstrom G., Backstrom T. (1997). The regional brain distribution of the neurosteroids pregnenolone and pregnenolone sulfate following intravenous infusion. J. Steroid Biochem. Mol. Biol..

[68] Asaba H., Hosoya K., Takanaga H., Ohtsuki S., Tamura E., Takizawa T., Terasaki T. (2000). Blood-brain barrier is involved in the efflux transport of a neuroactive steroid, dehydroepiandrosterone sulfate, via organic anion transporting polypeptide 2. J. Neurochem..

[69] Grube M., Hagen P., Jedlitschky G. (2018). Neurosteroid Transport in the Brain: Role of ABC and SLC Transporters. Front. Pharm..

[70] Schäfer A.M., Gilgen P.M., Spirgi C., Potterat O., Meyer Zu Schwabedissen H.E. (2021). Constituents of Passiflora incarnata, but Not of Valeriana officinalis, Interact with the Organic Anion Transporting Polypeptides (OATP)2B1 and OATP1A2. Planta Med..

[71] Visser T.J., Feingold K.R., Anawalt B., Boyce A., Chrousos G., de Herder W.W., Dhatariya K., Dungan K., Grossman A., Hershman J.M., Hofland J. (2000). Cellular Uptake of Thyroid Hormones. Endotext.

[72] Mullur R., Liu Y.Y., Brent G.A. (2014). Thyroid hormone regulation of metabolism. Physiol. Rev..

[73] Mayerl S., Visser T.J., Darras V.M., Horn S., Heuer H. (2012). Impact of Oatp1c1 deficiency on thyroid hormone metabolism and action in the mouse brain. Endocrinology.

[74] Sugiyama D., Kusuhara H., Taniguchi H., Ishikawa S., Nozaki Y., Aburatani H., Sugiyama Y. (2003). Functional characterization of rat brain-specific organic anion transporter (Oatp14) at the blood-brain barrier: High affinity transporter for thyroxine. J. Biol. Chem..

[75] Jansen J., Friesema E.C., Milici C., Visser T.J. (2005). Thyroid hormone transporters in health and disease. Thyroid.

[76] Meyer Zu Schwabedissen H.E., Ferreira C., Schaefer A.M., Oufir M., Seibert I., Hamburger M., Tirona R.G. (2018). Thyroid Hormones Are Transport Substrates and Transcriptional Regulators of Organic Anion Transporting Polypeptide 2B1. Mol. Pharm..

[77] Schwartz C.E., May M.M., Carpenter N.J., Rogers R.C., Martin J., Bialer M.G., Ward J., Sanabria J., Marsa S., Lewis J.A. (2005). Allan-Herndon-Dudley syndrome and the monocarboxylate transporter 8 (MCT8) gene. Am. J. Hum. Genet..

[78] van der Deure W.M., Hansen P.S., Peeters R.P., Kyvik K.O., Friesema E.C., Hegedus L., Visser T.J. (2008). Thyroid hormone transport and metabolism by organic anion transporter 1C1 and consequences of genetic variation. Endocrinology.

[79] Stromme P., Groeneweg S., Lima de Souza E.C., Zevenbergen C., Torgersbraten A., Holmgren A., Gurcan E., Meima M.E., Peeters R.P., Visser W.E. (2018). Mutated Thyroid Hormone Transporter OATP1C1 Associates with Severe Brain Hypometabolism and Juvenile Neurodegeneration. Thyroid.

[80] Kobayashi D., Nozawa T., Imai K., Nezu J., Tsuji A., Tamai I. (2003). Involvement of human organic anion transporting polypeptide OATP-B (SLC21A9) in pH-dependent transport across intestinal apical membrane. J. Pharm. Exp. Ther..

[81] Keiser M., Kaltheuner L., Wildberg C., Muller J., Grube M., Partecke L.I., Heidecke C.D., Oswald S. (2017). The Organic Anion-Transporting Peptide 2B1 Is Localized in the Basolateral Membrane of the Human Jejunum and Caco-2 Monolayers. J. Pharm. Sci..

[82] Ferreira C., Hagen P., Stern M., Hussner J., Zimmermann U., Grube M., Meyer Zu Schwabedissen H.E. (2018). The scaffold protein PDZK1 modulates expression and function of the organic anion transporting polypeptide 2B1. Eur. J. Pharm. Sci..

[83] St-Pierre M.V., Hagenbuch B., Ugele B., Meier P.J., Stallmach T. (2002). Characterization of an organic anion-transporting polypeptide (OATP-B) in human placenta. J. Clin. Endocrinol. Metab..

[84] Yang Z.Z., Li L., Wang L., Xu M.C., An S., Jiang C., Gu J.K., Wang Z.J., Yu L.S., Zeng S. (2016). siRNA capsulated brain-targeted nanoparticles specifically knock down OATP2B1 in mice: A mechanism for acute morphine tolerance suppression. Sci. Rep..

[85] Brecht K., Schäfer A.M., Meyer Zu Schwabedissen H.E. (2020). Uptake Transporters of the SLC21, SLC22A, and SLC15A Families in Anticancer Therapy-Modulators of Cellular Entry or Pharmacokinetics?. Cancers.

[86] Fracassi A., Marangoni M., Rosso P., Pallottini V., Fioramonti M., Siteni S., Segatto M. (2019). Statins and the Brain: More than Lipid Lowering Agents?. Curr. Neuropharmacol..

[87] Johnson-Anuna L.N., Eckert G.P., Keller J.H., Igbavboa U., Franke C., Fechner T., Schubert-Zsilavecz M., Karas M., Muller W.E., Wood W.G. (2005). Chronic administration of statins alters multiple gene expression patterns in mouse cerebral cortex. J. Pharm. Exp. Ther..

[88] Grube M., Kock K., Oswald S., Draber K., Meissner K., Eckel L., Bohm M., Felix S.B., Vogelgesang S., Jedlitschky G. (2006). Organic anion transporting polypeptide 2B1 is a high-affinity transporter for atorvastatin and is expressed in the human heart. Clin. Pharm. Ther..

[89] Varma M.V., Rotter C.J., Chupka J., Whalen K.M., Duignan D.B., Feng B., Litchfield J., Goosen T.C., El-Kattan A.F. (2011). pH-sensitive interaction of HMG-CoA reductase inhibitors (statins) with organic anion transporting polypeptide 2B1. Mol. Pharm..

[90] Shirasaka Y., Suzuki K., Shichiri M., Nakanishi T., Tamai I. (2011). Intestinal absorption of HMG-CoA reductase inhibitor pitavastatin mediated by organic anion transporting polypeptide and P-glycoprotein/multidrug resistance 1. Drug Metab. Pharm..

[91] Navratilova L., Ramos Mandikova J., Pavek P., Mladenka P., Trejtnar F. (2018). Honey flavonoids inhibit hOATP2B1 and hOATP1A2 transporters and hOATP-mediated rosuvastatin cell uptake in vitro. Xenobiotica.

[92] Endres M., Laufs U., Huang Z., Nakamura T., Huang P., Moskowitz M.A., Liao J.K. (1998). Stroke protection by 3-hydroxy-3-methylglutaryl (HMG)-CoA reductase inhibitors mediated by endothelial nitric oxide synthase. Proc. Natl. Acad. Sci. USA.

[93] Schultz B.G., Patten D.K., Berlau D.J. (2018). The role of statins in both cognitive impairment and protection against dementia: A tale of two mechanisms. Transl. Neurodegener..

[94] Abdullahi W., Brzica H., Hirsch N.A., Reilly B.G., Ronaldson P.T. (2018). Functional Expression of Organic Anion Transporting Polypeptide 1a4 Is Regulated by Transforming Growth Factor-beta/Activin Receptor-like Kinase 1 Signaling at the Blood-Brain Barrier. Mol. Pharm..

[95] Ose A., Kusuhara H., Endo C., Tohyama K., Miyajima M., Kitamura S., Sugiyama Y. (2010). Functional characterization of mouse organic anion transporting peptide 1a4 in the uptake and efflux of drugs across the blood-brain barrier. Drug Metab. Dispos..

[96] Sano Y., Mizuno T., Mochizuki T., Uchida Y., Umetsu M., Terasaki T., Kusuhara H. (2018). Evaluation of Organic Anion Transporter 1A2-knock-in Mice as a Model of Human Blood-brain Barrier. Drug Metab. Dispos..

